# Intraperitoneal Chemotherapy as Adjuvant or Perioperative Chemotherapy for Patients with Type 4 Scirrhous Gastric Cancer: PHOENIX-GC2 Trial

**DOI:** 10.3390/jcm10235666

**Published:** 2021-11-30

**Authors:** Hironori Ishigami, Yasushi Tsuji, Hisashi Shinohara, Yasuhiro Kodera, Mitsuro Kanda, Hiroshi Yabusaki, Seiji Ito, Motohiro Imano, Hiroharu Yamashita, Akio Hidemura, Hironori Yamaguchi, Takeo Fukagawa, Koji Oba, Joji Kitayama, Yasuyuki Seto

**Affiliations:** 1 Department of Chemotherapy, The University of Tokyo Hospital, Tokyo 113-8655, Japan; 2 Department of Gastrointestinal Surgery, Graduate School of Medicine, The University of Tokyo, Tokyo 113-8655, Japan; seto-tky@umin.ac.jp; 3 Department of Medical Oncology, Tonan Hospital, Sapporo 060-0004, Japan; ytsuji@tonan.gr.jp; 4 Department of Gastroenterological Surgery, Division of Upper GI, Hyogo College of Medicine, Nishinomiya 663-8507, Japan; shinohara@hyo-med.ac.jp; 5 Department of Gastroenterological Surgery, Nagoya University Graduate School of Medicine, Nagoya 466-8550, Japan; ykodera@med.nagoya-u.ac.jp (Y.K.); m-kanda@med.nagoya-u.ac.jp (M.K.); 6 Department of Gastroenterological Surgery, Niigata Cancer Center Hospital, Niigata 951-8566, Japan; yabu@niigata-cc.jp; 7 Department of Gastrointestinal Surgery, Aichi Cancer Center Hospital, Nagoya 464-8681, Japan; seito@aichi-cc.jp; 8 Department of Surgery, Kindai University Faculty of Medicine, Osakasayama 589-8511, Japan; imano@med.kindai.ac.jp; 9 Department of Digestive Surgery, Nihon University School of Medicine, Tokyo 101-8309, Japan; hyamashi-tky@umin.net; 10 Department of Surgery, Kanto Rosai Hospital, Kawasaki 211-8510, Japan; hidemura@kantoh.johas.go.jp; 11 Department of Clinical Oncology, Jichi Medical University Hospital, Shimotsuke 329-0498, Japan; yamaguchi@jichi.ac.jp; 12 Department of Surgery, Teikyo University School of Medicine, Tokyo 173-8605, Japan; tfukagaw@yahoo.co.jp; 13 Interfaculty Initiative in Information Studies, Department of Biostatistics, School of Public Health, Graduate School of Medicine, The University of Tokyo, Tokyo 113-0033, Japan; oba@epistat.m.u-tokyo.ac.jp; 14 Clinical Research Center, Department of Surgery, Jichi Medical University, Shimotsuke 329-0498, Japan; kitayama@jichi.ac.jp

**Keywords:** type 4 gastric cancer, peritoneal metastasis, adjuvant chemotherapy, perioperative chemotherapy, intraperitoneal chemotherapy, randomized clinical trial

## Abstract

The prognosis of patients with type 4 scirrhous gastric cancer remains poor due to a high risk of peritoneal metastasis. We have previously developed combined chemotherapy regimens of intraperitoneal (IP) paclitaxel (PTX) and systemic chemotherapy, and promising clinical efficacy was reported in gastric cancer with peritoneal metastasis. Herein, a randomized, phase III study is proposed to verify the efficacy of IP PTX to prevent peritoneal recurrence. Gastric cancer patients with type 4 tumors and without apparent distant metastasis, including peritoneal metastasis, will be randomized for standard systemic chemotherapy or combined IP and systemic chemotherapy based on peritoneal lavage cytology findings. Those with negative peritoneal cytology will receive radical gastrectomy and adjuvant chemotherapy of S-1 plus docetaxel (control arm), or S-1 plus intravenous and IP PTX (experimental arm). Those with positive peritoneal cytology will receive three courses of S-1 plus oxaliplatin (control arm), or S-1 plus oxaliplatin and IP PTX (experimental arm). Subsequently, they undergo gastrectomy and receive postoperative chemotherapy of S-1 plus docetaxel (control arm), or S-1 plus intravenous and IP PTX (experimental arm). The primary endpoint is disease free survival after a 3-year follow-up period. Secondary endpoints are overall survival, survival without peritoneal metastasis, safety, completion rate, curative resection rate, and histological response of preoperative chemotherapy. A total of 300 patients are to be enrolled.

## 1. Introduction

### 1.1. Characteristics of Type 4 Gastric Cancer

Type 4 (diffuse infiltrative) gastric cancer is defined as “tumors without marked ulceration or raised margins, the gastric wall is thickened and indurated, and the margin is unclear” based on Borrmann classification [1]. Type 4 tumors predominantly comprise poorly differentiated adenocarcinomas and signet-ring cell carcinomas and are characterized by abundant stromal fibrosis.

According to the nationwide registry of the Japanese Gastric Cancer Association, type 4 gastric cancer accounts for 14% of resected advanced gastric cancer cases. The 5-year survival rate of surgically resected type 4 gastric cancer was 21.4%, which is much lower than those of other macroscopic types of advanced gastric cancer (46.7–63.7%) [2]. This is mainly owing to the high incidence of peritoneal recurrence after surgery. Despite its unique clinical characteristics, only a few clinical studies have been conducted on type 4 gastric cancer. Thus, it is treated similarly as whole advanced gastric cancer [3,4,5,6,7,8,9,10,11,12].

### 1.2. Treatment of Locally Advanced Gastric Cancer

The Japanese gastric cancer treatment guidelines recommend gastrectomy with D2 lymph node dissection and postoperative adjuvant chemotherapy for locally advanced gastric cancer [3]. Based on the results of phase III clinical trials, S-1 (tegafur/gimeracil/oteracil) monotherapy is recommended for pStage II patients and S-1/docetaxel (DOC) and capecitabine/oxaliplatin (L-OHP) for pStage III patients [4,5,6].

The efficacy of preoperative neoadjuvant chemotherapy (NAC) has been investigated in patients with a high risk of postoperative recurrence to further improve survival. S-1/cisplatin (CDDP) resulted in a high response rate of 65% and a 5-year survival of 53% for patients with extensive lymph node metastasis [7]. However, this regimen failed to demonstrate survival benefits for patients with type 4 or large type 3 gastric cancer [8]. Meanwhile, S-1/L-OHP (SOX) resulted in an adequate feasibility and a high pathological response rate of 85.7% [9] and is currently being investigated in a phase III trial in Japan. A Korean phase III trial reported a superior progression-free survival rate with DOC/L-OHP/S-1, albeit a similar overall survival (OS) rate [10]. Consequently, the efficacy of NAC remains inconclusive in Asia.

Meanwhile, in the Western countries, different treatments have been developed. The efficacy of perioperative chemotherapy with fluorouracil/leucovorin/L-OHP/DOC (FLOT) is superior to that of epirubicin/CDDP/fluorouracil (ECF) [11] and is the standard treatment in Europe. Postoperative adjuvant chemoradiotherapy showed superiority over surgery alone [12] and is regarded as the standard treatment option in the United States.

### 1.3. Treatment of Gastric Cancer with Positive Peritoneal Lavage Cytology

Even without macroscopic peritoneal metastasis (P0), patients with positive peritoneal cytology (CY1) have a significantly poorer prognosis than those with negative peritoneal cytology (CY0) [2]. The standard treatment for P0CY1 patients has yet to be established because of the lack of high-level evidence [3]. In practice, the following kinds of treatment are performed: chemotherapy first with S-1/CDDP, SOX, or S-1/DOC [13,14,15], followed by gastrectomy after response; and gastrectomy followed by postoperative S-1 monotherapy or doublet regimens [16]. A recent multi-institutional retrospective study in Japan reported that the initial chemotherapy and initial surgery groups had similar survival rates, and that the patients achieving conversion to CY0 P0 had a favorable survival [17]. At present, a promising multidisciplinary treatment is a combination of preoperative chemotherapy with SOX, gastrectomy, and postoperative treatment with S-1/DOC. SOX showed adequate feasibility as NAC [9] and favorable efficacy for P1 patients in the subgroup analysis of a phase III study [14]. S-1/DOC demonstrated efficacy in patients with unresectable advanced and stage III gastric cancer [5,15].

### 1.4. Intraperitoneal Chemotherapy for Gastric Cancer

The peak drug concentration and sustained duration in the target tumor are important factors to determine the effect of anticancer drugs. Intraperitoneal (IP) administration delivers drugs at high concentrations directly to the peritoneal tumors and free cancer cells in the peritoneal cavity. Paclitaxel (PTX) is slowly absorbed through the peritoneum via the lymphatic system after IP administration owing to its lipophilicity and high molecular weight. In a phase I study of IP PTX in ovarian cancer, significant concentrations persisted in the peritoneal cavity for more than 24 to 48 h. The peak levels and area under the concentration time curve (AUC) in the peritoneal cavity were 1000 times higher than those in the plasma. The dose was escalated up to 200 mg/m^2^, and the dose-limiting toxicity was severe abdominal pain at doses more than 175 mg/m^2^ [18]. IP administered drug infiltrates the peritoneal tumor from the surface into the inside and is effective in the peripheral zones of the tumor. However, a sufficient amount of drug is not delivered to the central zone of the peritoneal tumor, the primary tumor in the stomach, and metastasis to lymph nodes or other organs. Therefore, the administration of IP PTX should be frequently repeated with systemic chemotherapy to obtain the maximum effect.

In ovarian cancer, the efficacy of combined IP PTX and systemic chemotherapy was verified in clinical trials conducted in Western countries [19]. Thus, it is now a recommended treatment option according to the National Cancer Institute guidelines. In gastric cancer, the effectiveness and safety of IP PTX and IP DOC were first reported by Fushida et al. [20] in 2005. Since then, our study group named the Japan Intraperitoneal Chemotherapy Study Group has designed several combination chemotherapy regimens including IP PTX or DOC and performed clinical trials for the ministerial approval of the new route of administration under the advanced medical system in Japan [21,22,23,24,25,26,27].

#### 1.4.1. S-1/PTX+IP PTX Regimen

We designed a regimen combining weekly IP PTX with S-1/PTX, a candidate regimen for unresectable or recurrent gastric cancer in 2006. The recommended dose of IP PTX was determined to be 20 mg/m^2^ in a dose escalation study, with dose limiting toxicities (DLTs) of febrile neutropenia and diarrhea. The intraperitoneal and serum PTX concentrations were above the effective level for over 72 and 48 h, respectively [21]. In a phase II study, the 1-year OS rate was 78%, and the negative conversion rate on peritoneal cytology was 86% [22]. Subsequent phase II studies under the advanced medical system reported 1-year OS rates of 77% in P1 patients [23] and 84% in P0CY1 patients [24]. The phase III PHOENIX-GC trial in P1 patients narrowly failed to show the statistical superiority of this regimen over S-1/CDDP (median survival time 17.7 vs. 15.2 months, *p* = 0.080; hazard ratio [HR] 0.72; 95% confidence interval [CI] 0.49–1.04). However, the exploratory analysis adjusting for the baseline imbalance in the amount of ascites between the arms suggested clinical benefits (HR 0.59, 95% CI 0.39–0.87). Moreover, the 3-year OS rate was 21.9% (95% CI 14.9–29.9) in the IP arm and 6.0% (95% CI 1.6–14.9) in the reference arm after an additional 1-year follow-up. Safety was confirmed, and adverse events (grade 3/4), including neutropenia (50%), leucopenia (25%), anemia (13%), and anorexia (10%), were reported. IP port-related complications were infection (3%) and catheter obstruction (3%) [25].

#### 1.4.2. SOX+IP PTX Regimen

SOX regimen demonstrated non-inferiority to S-1/CDDP regimen [14] in 2015 and is now a recommended regimen for unresectable or recurrent gastric cancer in Japan [3]. Therefore, we designed a regimen combining weekly IP PTX with SOX to intensify the systematic effects against the primary tumor and metastasis to lymph nodes or other organ sites. In a phase I study, the dose of IP PTX was increased from 20 to 40 mg/m^2^ without developing any DLT, and the recommended dose was determined to be 40 mg/m^2^ [26]. The phase II study showed a 1-year OS rate of 72% and a negative conversion rate on peritoneal cytology of 84%. Frequent adverse events (grade 3/4) were neutropenia (50%), leucopenia (28%), anemia (18%), and anorexia (12%) [27]. Both the efficacy and safety of SOX+IP PTX regimen were similar to those of S-1/PTX+IP PTX regimen.

### 1.5. Intraperitoneal Chemotherapy Combined with Gastrectomy

In a preliminary study of combined IP and systemic chemotherapy in patients with gastric cancer with serosal invasion, only 1 of 10 patients developed peritoneal recurrence, and the 5-year OS rate was 88% [28]. A multicenter phase II study of the same population showed the safety of perioperative combined IP and systemic chemotherapy and gastrectomy. An excellent pathological response of 68% was reported, and the survival will be evaluated in the near future [29]. Meanwhile, IP PTX was compared with intravenous PTX as monotherapy in the adjuvant setting, and the negative results possibly suggested the importance to combine systemic chemotherapy with IP PTX [30]. Additionally, the safety of gastrectomy after chemotherapy and that of postoperative combination chemotherapy were confirmed in a retrospective study of surgery after response to combined IP and systemic chemotherapy in CY1/P1 patients [31].

## 2. Methods

### 2.1. Objective

This study aims to assess and compare the efficacy and safety of combined IP and systemic chemotherapy with those of standard systemic chemotherapy as adjuvant or perioperative chemotherapy in patients with type 4 gastric cancer.

### 2.2. Eligibility Criteria

Gastric cancer patients with type 4 tumors and without clinically apparent distant metastasis, including peritoneal metastasis, are enrolled. The inclusion and exclusion criteria are summarized in Table 1.

### 2.3. Endpoints

The primary endpoint is disease-free survival (DFS). DFS was demonstrated as an acceptable surrogate endpoint for OS in a meta-analysis of clinical trials of adjuvant chemotherapy for gastric cancer [32]. 

Secondary endpoints are OS, peritoneal recurrence-free survival, the incidence of adverse events in all patients, completion rate of preoperative chemotherapy, curative resection rate, and histological response rate in CY0 patients.

### 2.4. Study Design and Procudure

This is a multicenter, open-label, randomized, parallel-group comparison, confirmatory clinical study. The study is planned to be performed in 7 years (from June 2020 to May 2027), comprising 3 years of patient enrollment from 40 hospitals throughout Japan and 3 years of follow-up period.

**Figure 1:**
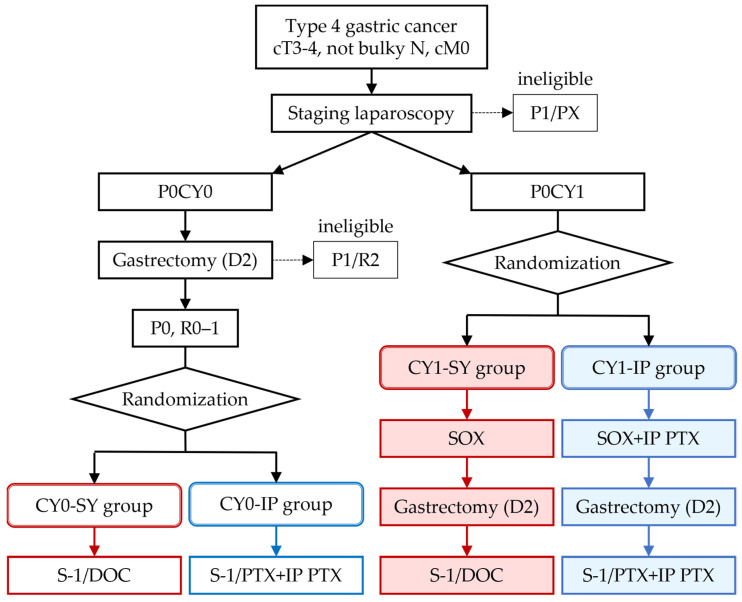
Flow of the study. P0, no peritoneal metastasis; P1, peritoneal metastasis; PX, peritoneal metastasis is unknown; CY0, peritoneal cytology negative for carcinoma cells; CY1, peritoneal cytology positive for carcinoma cells; R0, no residual tumor after surgery; R1, microscopic residual tumor (positive resection margin or CY1); R2, macroscopic residual tumor; SY group, systemic chemotherapy group; IP group, combined IP and systemic chemotherapy group. The red boxes and the blue boxes represent the group names and the treatments of the systemic chemotherapy groups and the combined IP and systemic chemotherapy groups, respectively.

The flow of the study is shown in Figure 1. Patients who fulfill the eligibility criteria except for inclusion criteria 12 (P0) and 13 (R0–1) are provisionally registered and undergo staging laparoscopy and intraoperative peritoneal lavage cytology. Patients are and will be randomized to receive either standard systemic chemotherapy or combined IP and systemic chemotherapy based on the results of peritoneal lavage cytology. Randomization is stratified by institution and clinical lymph node metastasis (cN0/cN1/cN2 by location according to the former Japanese Classification of Gastric Carcinoma [33]). Patients allocated to the investigational group will be implanted with an IP port (Bard Peritoneal Titanium port; Becton Dickinson, Covington, GA, USA) in the subcutaneous space of the abdomen.

CY0 patients will receive radical gastrectomy and will be randomized when macroscopic curative resection (R0–1) is achieved. Patients will receive adjuvant chemotherapy of S-1/DOC in the control group and S-1/PTX plus IP PTX in the investigational group.

CY1 patients will first receive three courses of chemotherapy of SOX in the control group and SOX plus IP PTX in the investigational group. Subsequently, they will undergo gastrectomy and receive postoperative chemotherapy of S-1/DOC in the control group and S-1/PTX plus IP PTX in the investigational group.

The number of courses of chemotherapy is three before gastrectomy and seven after gastrectomy based on the recent clinical trials of NAC and adjuvant chemotherapy [5,9]. Administration of DOC or PTX in the first course after gastrectomy is omitted for better feasibility. Overall, the regimens in the investigational groups correspond to the combinations of IP PTX with the regimens in the control groups, with the replacement of DOC with PTX in the postoperative treatment.

### 2.5. Chemotherapy Regimens

**Figure 2:**
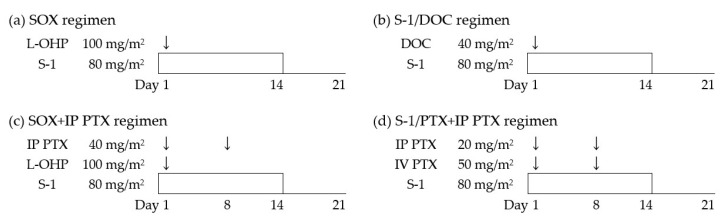
Treatment schedule and doses of chemotherapy regimens. (**a**) Preoperative chemotherapy for the CY1-SY group, (**b**) postoperative chemotherapy for the CY0-SY and CY1-SY groups, (**c**) preoperative chemotherapy for the CY1-IP group, and (**d**) postoperative chemotherapy for the CY0-IP and CY1-IP groups.

The treatment schedule and doses of chemotherapy regimens are shown in Figure 2. S-1 is administered orally twice daily at 80 mg/m^2^/day according to the body surface area (BSA): 80 mg/day for BSA < 1.25 m^2^, 100 mg/day for BSA ≥ 1.25 to <1.5 m^2^, and 120 mg/day for BSA ≥ 1.5 m^2^ on days 1 to 14 of every 3-week cycle. Intravenous administration of DOC, PTX, and L-OHP is performed according to the standard procedure described in the package inserts. For IP administration, PTX in 500 mL of normal saline is administered via an implanted port for 1 h, after the administration of 500 mL of normal saline. 

### 2.6. Sample Size Calculation and Statistical Considerations

This study was designed to verify the superiority of combined IP and systemic chemotherapy (IP group; CY0-IP and CY1-IP groups) over systemic chemotherapy (SY group; CY0-SY and CY1-SY groups) with DFS as the primary endpoint. The hypotheses are the following: 3-year DFS in SY group of 50% (60% in CY0-SY group, 30% in CY1-SY group [8]); a ratio of CY0 patients to CY1 patients of 2:1; hazard ratios of 0.64 for both CY0-IP to CY0-SY and CY1-IP to CY1-SY; a dropout rate of 10%; a follow-up period of 3 years; and a one-sided significant level of 2.5%. To achieve a power of 80% under these hypotheses, the required number of events and patients was calculated as 157 and 300, respectively, using the Lakatos method [34] in SAS ver. 9.4 (SAS Institute, Cary, NC, USA) Power Procedure.

The efficacy analysis will be performed for all randomized patients. DFS was defined from randomization to first evidence of recurrence, second primary malignancy, or death. The difference between the IP and SY groups will be assessed using the stratified log-rank test (stratified by CY0/CY1 and other allocation factor) after evaluation of the interaction between CY0/CY1 and IP/SY group. Survival curves are estimated using the Kaplan–Meier method, and hazard ratios are estimated using a stratified Cox regression model. One interim analysis is planned to re-estimate the sample size accompanied with futility stopping based on a conditional power after 79 events (50% of the planned events) have been observed.

## 3. Future Perspective

We hope that this trial will demonstrate the efficacy of combined IP and systemic chemotherapy for type 4 gastric cancer, and that this treatment will save as many lives as possible as the standard of care in Japan and worldwide.

## Figures and Tables

**Table 1:** Inclusion and exclusion criteria of the PHOENIX-GC2 trial.

Inclusion criteria (1) Pathologically confirmed common-type gastric adenocarcinoma ^1^ (2) Macroscopically type 4 (diffuse infiltrating type) tumor (3) Tumor invasion of the subserosa, serosa, or adjacent structures suggested on diagnostic imaging (cT3–4) (4) No bulky lymph node metastasis detected on CT (5) No apparent distant metastasis detected on diagnostic imaging (cM0) (6) Age 20–75 years (7) ECOG performance status; 0 or 1 (8) No previous chemotherapy or radiotherapy (9) Adequate organ functions as shown below: neutrophil count ≥ 1500/mm^3^, hemoglobin ≥ 8.0 g/dl, platelet count ≥ 100,000/mm^3^, AST and ALT ≤ 100 U/L, T. bilirubin ≤ 2.0 mg/dl, creatinine clearance ≥ 50 mL/min (10) Possible oral intake (11) Written informed consent from the patient (12) No peritoneal metastasis by staging laparoscopy (P0)	(13) Either of the following results of peritoneal lavage cytology and residual tumor status after gastrectomy: Negative peritoneal lavage cytology (CY0) and macroscopic curative resection performed (R0–1) Positive peritoneal lavage cytology (CY1) and macroscopic curative resection considered achievable (R0–1) Exclusion criteria (1) Synchronous double cancer excluding lesions equal to intraepithelial or intramucosal cancer (2) Serious complications, including interstitial pneumonia, pulmonary fibrosis, uncontrollable diabetes, poor controlled hypertension, cardiac failure, renal failure, hepatic cirrhosis, and hepatic failure (3) Contraindications to S-1, docetaxel, paclitaxel, or oxaliplatin (4) Pregnant or planning pregnancy (5) Judged by investigators as ineligible for this study

Inclusion criteria 1–11 and all exclusion criteria should be fulfilled before provisional registration. Inclusion criteria 12–13 should be fulfilled at the time of registration during staging laparoscopy or gastrectomy. ^1^ The common-type gastric adenocarcinoma consists of papillary adenocarcinoma, tubular adenocarcinoma, poorly differentiated adenocarcinoma (poorly cohesive carcinoma), signet-ring cell carcinoma, and mucinous adenocarcinoma [1].
